# Heart failure with preserved ejection fraction management: a systematic review of clinical practice guidelines and recommendations

**DOI:** 10.1093/ehjqcco/qcae053

**Published:** 2024-06-25

**Authors:** Adil Mahmood, Eamon Dhall, Christopher P Primus, Angela Gallagher, Rosita Zakeri, Selma F Mohammed, Anwar A Chahal, Fabrizio Ricci, Nay Aung, Mohammed Y Khanji

**Affiliations:** William Harvey Research Institute, NIHR Barts Biomedical Research Centre, Queen Mary University of London, Charterhouse Square, London EC1M 6BQ, UK; Newham University Hospital, Barts Health NHS Trust, Glen Road, London E13 8SL, UK; Newham University Hospital, Barts Health NHS Trust, Glen Road, London E13 8SL, UK; Barts Heart Centre, St Bartholomew's Hospital, Barts Health NHS Trust, West Smithfield, London EC1A 7BE, UK; Newham University Hospital, Barts Health NHS Trust, Glen Road, London E13 8SL, UK; Barts Heart Centre, St Bartholomew's Hospital, Barts Health NHS Trust, West Smithfield, London EC1A 7BE, UK; School of Cardiovascular Medicine & Sciences, James Black Centre, King's College London, 125 Coldharbour Lane, London SE5 9NU, UK; Department of Cardiology, Creighton University School of Medicine, Omaha, NE 68124, USA; William Harvey Research Institute, NIHR Barts Biomedical Research Centre, Queen Mary University of London, Charterhouse Square, London EC1M 6BQ, UK; Barts Heart Centre, St Bartholomew's Hospital, Barts Health NHS Trust, West Smithfield, London EC1A 7BE, UK; Department of Cardiovascular Medicine, Mayo Clinic, 200 First Str, SW Rochester, MN 55905, USA; Center for Inherited Cardiovascular Diseases, Department of Cardiology, WellSpan Health, 30 Monument Rd, York, PA 17403, USA; Department of Neuroscience, Imaging and Clinical Sciences, “G. d'Annunzio” University of Chieti-Pescara, Via dei Vestini 33, 66100 Chieti, Italy; University Cardiology Division, SS Annunziata Polyclinic University Hospital, Via dei Vestini 5, 66100 Chieti, Italy; Department of Clinical Sciences, Lund University, Jan Waldenströms Gata 35, 21428 Malmö, Sweden; William Harvey Research Institute, NIHR Barts Biomedical Research Centre, Queen Mary University of London, Charterhouse Square, London EC1M 6BQ, UK; Barts Heart Centre, St Bartholomew's Hospital, Barts Health NHS Trust, West Smithfield, London EC1A 7BE, UK; William Harvey Research Institute, NIHR Barts Biomedical Research Centre, Queen Mary University of London, Charterhouse Square, London EC1M 6BQ, UK; Newham University Hospital, Barts Health NHS Trust, Glen Road, London E13 8SL, UK; Barts Heart Centre, St Bartholomew's Hospital, Barts Health NHS Trust, West Smithfield, London EC1A 7BE, UK

**Keywords:** Heart failure, Heart failure with preserved ejection fraction, Diastolic dysfunction, Guideline recommendations, Systematic review

## Abstract

Multiple guidelines exist for the diagnosis and management of heart failure with preserved ejection fraction (HFpEF). We systematically reviewed current guidelines and recommendations, developed by national and international medical organizations, on the management of HFpEF in adults to aid clinical decision-making. We searched MEDLINE and EMBASE on 28 February 2024 for publications over the last 10 years as well as websites of organizations relevant to guideline development. Of the 10 guidelines and recommendations retrieved, 7 showed considerable rigour of development and were subsequently retained for analysis. There was consensus on the definition of HFpEF and the diagnostic role of serum natriuretic peptides and resting transthoracic echocardiography. Discrepancies were identified in the thresholds of serum natriuretic peptides and transthoracic echocardiography parameters used to diagnose HFpEF. There was agreement on the general pharmacological and supportive management of acute and chronic HFpEF. However, differences exist in strategies to identify and address specific phenotypes. Contemporary guidelines for HFpEF management agree on measures to avoid its development and the consideration of cardiac transplantation in advanced diseases. There were discrepancies in recommended frequency of surveillance for patients with HFpEF and sparse recommendations on screening for HFpEF in the general population, use of diagnostic scoring systems, and the role of newly emerging therapies.

Key learning points **What is already known:**

- Numerous clinical practice guidelines developed by national and international medical organizations on the management of heart failure with preserved ejection fraction have been published in recent years, following breakthrough randomized controlled trial data on emerging therapies.

**What this study adds:**

- To our knowledge, this is the first systematic review of current guidelines and recommendations on the management of heart failure with preserved ejection fraction to assess areas of agreement and disagreement as well as gaps in evidence. We anticipate this will improve clinical decision-making and guide future research.

## Introduction

Heart failure (HF) is associated with significant morbidity and mortality with a worse prognosis than certain common cancers.^1^ It exerts an increasing economic burden on society,^2^ with incidence continuing to rise with evolving population demographics.^3^ Indeed, recent estimates indicate the absolute number of patients living with HF has risen by 23% from 2002 to 2014.^4^ HF is broadly divided into three main subtypes: (i) HF with reduced ejection fraction (HFrEF); (ii) HF with mildly reduced ejection fraction (HFmrEF); and (iii) HF with preserved ejection (HFpEF). HFpEF is a condition characterized by clinical symptoms and signs of HF in the setting of normal or near-normal left ventricular (LV) ejection fraction (LVEF) attributed to raised LV filling pressures at rest or on exertion. Approximately half of all HF patients suffer from HFpEF,^5^ and it is more commonly seen in individuals with cardiometabolic diseases, with incidence climbing with advancing age^.6^ With an ageing population, coupled with a rise in obesity and diabetes, the burden of this disease is projected to become of even greater significance for population health.^7^

Given the longstanding lack of disease-modifying therapies, management of HFpEF has largely been focused on symptom improvement, reducing congestion with diuretics, and treating cardiovascular and non-cardiovascular comorbidities. However, breakthrough randomized controlled trial data on the use of sodium—glucose co-transporter 2 (SGLT2) inhibitors in patients with HFpEF demonstrated reduced composite of cardiovascular death or HF hospitalizations.^8,9^ Following these trials, updated clinical practice guidelines on HFpEF have been published in recent years. Therefore, our objective was to perform a systematic review of current guidelines and recommendations from professional organizations on the diagnosis and management of HFpEF in order to assess consensus and identify discrepancies to guide future research.

## Methods

### Data sources and searches

We conducted a systematic review of English language clinical practice guidelines and recommendations for the management of HFpEF in adults. We searched MEDLINE and EMBASE on 28 February 2024 for guidelines published in the last 10 years. We also searched websites of organizations relevant to guideline development (Supplementary material online, *Appendix Table 1*). This systematic review was planned, conducted, and reported in agreement with the Preferred Reporting Items for Systematic Reviews and Meta-Analyses (PRISMA) recommendations.^10^

### Study selection

We included contemporary documents published by professional organizations which made specific recommendations for HFpEF diagnosis and management in adults, and met the Institute of Medicine's definition of a guideline. Recommendations concerning HFmrEF were not considered. If more than one guideline from the same organization existed, we considered the most recent one. Where the most recent guideline was only a focused update, we combined both guidelines. Consensus statements were not included, regardless of publication date, in favour of the most recent guideline. We developed a search syntax in collaboration with an academic librarian which served as a basis for the search strategy (Supplementary material online). Key search terms included: ‘heart failure with preserved ejection fraction’, ‘heart failure with normal ejection fraction’, ‘diastolic heart failure’, ‘recommendation’, and ‘guideline*’.

### Data extraction and quality assessment

Titles and abstracts were assessed by two independent reviewers (A.M. and E.D.) using Rayyan.ai (https://www.rayyan.ai/). Articles were excluded if both reviewers agreed they were ineligible. Discrepancies were resolved by consensus after discussion. Both reviewers performed the final selection for full data extraction.

We used the 23-item Appraisal of Guidelines for Research and Evaluation (AGREE) II instrument to determine the rigour of development for each guideline.^11^ Two reviewers (A.M. and E.D.) independently rated the items, conforming to the instructions of the AGREE II tool. The average rigour scores were obtained by expressing the sum of the individual scores as a percentage of the maximum possible score. Reproducibility of the two reviewers’ scores was good, with an interclass correlation of 0.84 (Supplementary material online, *Appendix Table 2*). Guidelines were ranked according to their scores. Editorial independence from the funding body, external funding, and disclosure of relationships with industry by individual guideline group members were also assessed.

### Data synthesis and analysis

Two reviewers (A.M. and E.D.) extracted all relevant recommendations from the guidelines that had an AGREE II score equal to or greater than 50%. A recommendation matrix was produced.

## Results

**Figure 1:**
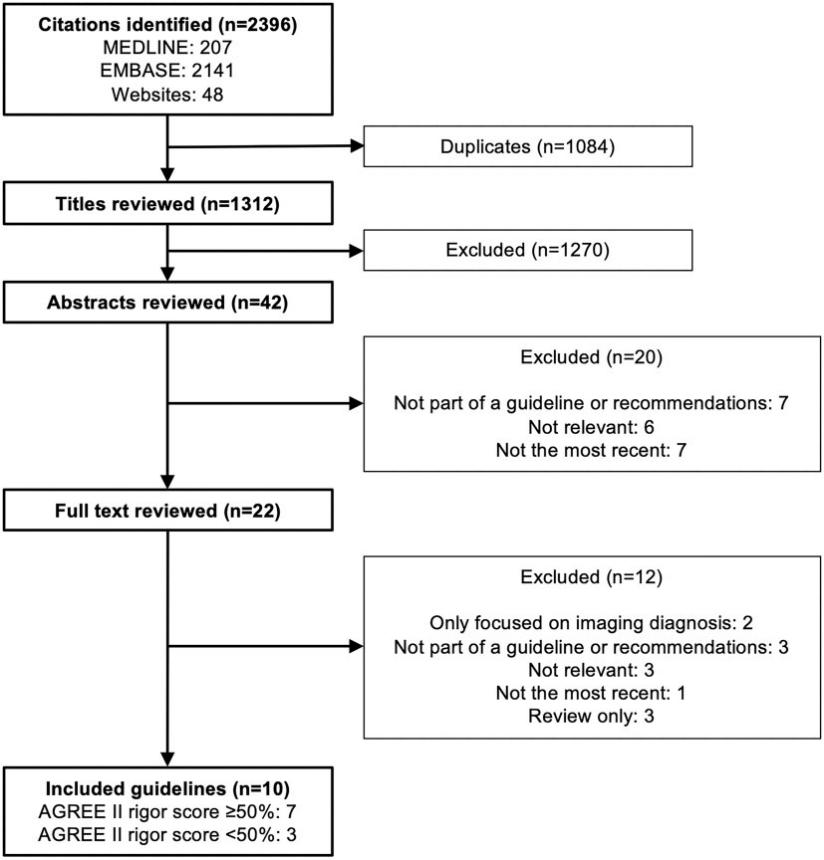
Summary of the guideline search and review process. The number of guidelines at each step is indicated. AGREE II, Appraisal of Guidelines for Research and Evaluation II.

We retrieved 2396 titles, of which 42 were potentially eligible. We retained 10 guidelines and recommendations on the management of HFpEF after review of the full manuscripts. Seven of the ten documents had a rigour score of ≥50% and form the object of this analysis (*Figure 1*). These were obtained from the following organization societies: American Heart Association/American College of Cardiology/Heart Failure Society of America (AHA/ACC/HFSA),^12^ National Heart Foundation of Australia/Cardiac Society of Australia and New Zealand (NHFA/CSANZ),^13^ Canadian Cardiovascular Society/Canadian Heart Failure Society (CCS/CHFS),^14^ European Society of Cardiology (ESC),^15,16^ Japanese Circulation Society/Japanese Heart Failure Society (JCS/JHFS),^17,18^ Saudi Heart Association (SHA),^19,20^ and National Institute for Health and Care Excellence (NICE) of the United Kingdom.^21,22^  *Table 1* summarizes the selected guidelines along with rigour scores and conflicts of interest.

**Table 1:** Summary of guideline recommendations for heart failure with preserved ejection fraction management

Organization society	National Institute for Health and Care Excellence (NICE)^14,15^	American Heart Association/American College of Cardiology/Heart Failure Society of America (AHA/ACC/HFSA)^16^	European Society of Cardiology (ESC)^17,18^	Canadian Cardiovascular Society/Canadian Heart Failure Society (CCS/CHFS)^19^	Saudi Heart Association (SHA)^20,21^	National Heart Foundation of Australia/Cardiac Society of Australia and New Zealand (NHFA/CSANZ)^22^	Japanese Circulation Society/Japanese Heart Failure Society (JCS/JHFS)^23,24^
Document type	Clinical practice guidelines	Clinical practice guidelines	Clinical practice guidelines	Clinical practice guidelines	Clinical practice guidelines	Clinical practice guidelines	Clinical practice guidelines
Region applied	United Kingdom	United States of America	Europe	Canada	Saudi Arabia	Australia and New Zealand	Japan
Year	2018 (update 2023)	2022	2021 (update 2023)	2017 (update 2020)	2019 (update 2022)	2018	2017 (update 2021)
AGREE II rigour score, %	88	85	72	69	68	66	55
Conflict of interest	EI, SCI^a,b^	EI, SCI^a,b^	EI, SCI^a^	Declaration no longer available	SCI^a^	SCI^a,b^	EI, SCI^a^
Methods used to evaluate evidence	Systematic review	Systematic review	Systematic review	Systematic review	Systematic review	Systematic review	Not specified
Methods used to formulate recommendations	Formal consensus	Formal consensus	Formal consensus	Formal consensus	Formal consensus	Formal consensus	Formal consensus
Consideration of costs	Cost considered in strength of recommendation	Information from studies on cost considered where available	Not reported	Not reported	Information from studies on cost considered where available	Information from studies on cost considered where available	Information from studies on cost considered where available
Definition of HFpEF	1. Symptoms of HF 2. LVEF ≥50% 3. Cardiac structure or function abnormalities and/or raised levels of NPs	1. Clinical signs and symptoms of HF 2. LVEF ≥50% 3. Evidence of spontaneous or provokable increased LV filling pressures including raised NPs, non-invasive and invasive hemodynamic measurements	1. Symptoms and signs of HF 2. LVEF ≥50% 3. Evidence of structural and/or functional cardiac abnormalities consistent with LV diastolic dysfunction/raised LV filling pressures, including raised NPs	1. Clinical symptoms and signs of reduced cardiac output and/or congestion at rest or with stress due to heart function 2. LVEF ≥50%	1. Evidence of congestive HF 2. LVEF ≥50% 3. LV diastolic dysfunction 4. Elevated NPs	1. Symptoms +/− signs of HF 2. LVEF ≥50% 3. Objective evidence of relevant structural heart disease and/or diastolic dysfunction with high filling pressures identified by either catheterization, TTE or elevated NPs	1. Clinical symptoms of HF 2. LVEF ≥50% 3. LV diastolic dysfunction
Key diagnostic tests	NT-proBNP—>400 ng/L TTE—assess diastolic function (no parameters or cut-offs provided)	NT-proBNP ≥125 pg/mL BNP ≥35 pg/mL Higher cut-offs recommended for AF or CKD TTE—parameters indicating raised LV filling pressures/structural heart disease: • Average E/e’ ≥15 • Septal e’ <7 cm/s • Lateral e’ <10 cm/s • TR velocity >2.8 m/s • PA systolic pressure >35 mmHg • LA volume index ≥29 mL/m^2^ • LV mass index >116 (male) or >95 (female) g/m^2^ • Relative wall thickness >0.42 • LV wall thickness ≥12 mm	NT-proBNP—>125 (SR) or >365 (AF) pg/mL BNP—>35 (SR) or >105 (AF) pg/mL Resting TTE—parameters indicating LV diastolic dysfunction/raised LV filling pressures: • LV mass index ≥95 (female) or ≥115 g/m2 (male) • LV relative wall thickness >0.42 • LA volume index >34 (SR) or >40 mL/m^2^ (AF) • E/e’ ratio at rest >9 • PA systolic pressure >35 mmHg • TR velocity at rest >2.8 m/s	NT-proBNP—>125 (ambulatory) or >300 (acute) pg/mL BNP—>50 (ambulatory) or >100 (acute) pg/mL TTE—include diastolic parameters e.g. transmitral and pulmonary venous flow patterns, or mitral annulus velocities (no cut-offs provided)	NT-proBNP—>125 pg/mL BNP—>35 pg/mL TTE—parameters to assess (no cut-offs provided): • LA volume index • LV mass index • LV wall thickness • Transmitral and tissue doppler indices • Longitudinal strain patterns • TR velocity • RV systolic function • TAPSE • RV systolic pressure	NT-proBNP—>450 (age <50 years) or > 900 (age 50–75 years) or >1800 (age >75 years) ng/L BNP—>400 ng/L Resting TTE—parameters indicating structural heart disease: • LV mass index >115 g/m^2^ (men) or >95 g/m^2^ (women) • LA volume index >34 mL/m^2^ **OR** diastolic dysfunction (≥3 abnormal suggestive): • Septal e’ <7 cm/s or lateral e’ <10 cm/s • Average E/e’ ratio >14 • LA volume index >34 mL/m^2^ • TR velocity >2.8 m/s	NT-proBNP—≥400 pg/mL BNP—≥100 pg/mL Mild HF cannot be ruled out if NT-proBNP 125–400 pg/mL or BNP 40–100 pg/mL Resting TTE—parameters of LV diastolic dysfunction (≥3 abnormal suggestive): • Average E/e’ >14 (septal E/e’ >15 or lateral E/e’ >13) • Septal e’ <7 cm/s or lateral e’ <10 cm/s • TR velocity >2.8 m/s • LA volume index >34 mL/m^2^
Other tests	Alternative imaging e.g. CMR, TOE or radionuclide angiography—consider if poor TTE images	CPET—delineate aetiology of dyspnoea, assess functional capacity and may guide prognosis **(1 C-LD)** Alternative imaging e.g. CMR, cardiac CT, radionuclide imaging—if TTE inadequate for LVEF assessment **(1 C-LD)** CMR—can be useful for diagnosis or management if known HF or cardiomyopathy **(2a B-NR)** Right heart catheterization—consider if persistent symptoms despite treatment **(2a C-EO)** Endomyocardial biopsy—consider if suspect specific cause for HF that would influence therapy **(2a B-NR)** Genetic screening and counselling**—**if genetic or inherited cardiomyopathy suspected **(1 B-NR)**	CPET—confirm reduction in exercise capacity and help differentiate cause of dyspnoea **(IIa C)** CMR—assess cardiac structure and function if TTE inadequate or further investigate aetiology **(I C)** Diastolic stress test—if equivocal resting TTE and laboratory markers **(IIb C)** Right heart catheterization—invasively measured PCWP ≥15 (at rest) or ≥25 (with exercise) mmHg or LVEDP ≥16 mmHg (at rest) are considered diagnostic **(IIb C)** Endomyocardial biopsy—consider in rapidly progressive HF where biopsy can detect suspected diagnosis **(IIa C)**	CMR—consider when TTE non-diagnostic or to help elucidate aetiology **(I LQE)**	CPET or stress echocardiography—consider in select cases to identify cause of dyspnoea **(IIb)** CMR—assess cardiac structure and function if TTE inadequate or further investigate aetiology **(I)** Right heart catheterization—workup for transplant if advanced HF **(I)** Endomyocardial biopsy—consider if suspect specific diagnosis that would influence therapy **(IIb)** Genetic testing—if familial cardiomyopathy suspected (**I–IIa** depending on suspected diagnosis)	CMR with LGE—consider to identify inflammatory and infiltrative cardiomyopathies if HF with increased and unexplained LV wall thickness **(I LQE)** PET or bone scintigraphy—consider to identify infiltrative cardiomyopathies if HF with increased and unexplained LV wall thickness **(II LQE)**	CPET—if cause of symptoms unclear or in workup to advanced treatments **(I B)** CMR—provides information on cardiac structure, function and aetiology if cannot assess with TTE **(I C)** ECG-gated SPECT—assess LV volume and LVEF if cannot assess with TTE **(IIa C)** Right heart catheterization**—**pulmonary artery pressure monitoring in select patients **(I C)** Endomyocardial biopsy—confirm specific diagnosis that affects HF treatment strategies **(IIa C)**
Prevention	Correct risk factors: • Salt and fluid restriction—not routinely advised; if needed, restrict fluids if dilutional hyponatraemia or reduce intake if high levels of salt and/or fluid consumption. Avoid salt substitutes containing potassium • Refer to dietetics if BMI <18.5 kg/m^2^ or weight loss advice if >30 kg/m^2^	Correct risk factors: • Healthy lifestyle modifications including physical activity, maintaining healthy weight, healthy diet and smoking cessation **(1 B-NR)** • Control hypertension—aim for <130/80 mmHg **(1 A)** • Statin if history of ACS **(1 A)** • SGLT2 inhibitors in patients with T2DM **(1 A)** Screening: • Population screening through NP monitoring **(2a B-R)** and risk scores **(2a B-NR)**	Correct risk factors: • Counselling against sedentary habit, obesity, cigarette smoking, and alcohol abuse **(I C)** • Treatment of hypertension **(I A)** • Treatment with statins in patients at high risk of CVD or with CVD **(I A)** • SGLT2 inhibitors in patients with diabetes at high risk of CVD or with CVD **(I A)** • SGLT2 inhibitors in patients with T2DM and CKD **(I A)** • Finerenone in patients with T2DM and CKD **(I A)**	Correct risk factors: • Healthy lifestyle modifications e.g. increased physical activity and healthy weight maintenance **(I MQE)** • Control hypertension—aim <140/90 mmHg, or SBP <130 mmHg in diabetes or high risk of CV event **(I MQE)** • Manage T2DM **(I MQE)**—metformin first line **(II MQE)**; consider empagliflozin if concurrent T2DM and established CVD **(II LQE)**	Correct risk factors: • Smoking cessation and treatment for those with alcohol excess **(I)** • Treatment of hypertension **(I)** • Treatment of obesity **(I)** • Statins if CVD or at risk of CVD **(I)** • SGLT2 inhibitor if T2DM with CVD or at high risk of CVD **(I)**	Correct risk factors: • Smoking cessation **(I LQE)** • Avoid excess alcohol **(I VLQE)** • Weight reduction **(I LQE)** • Regular physical activity **(I LQE)** • Treatment of hypertension and hyperlipidaemia **(I HQE)** • SGLT2 inhibitors in T2DM and CVD with insufficient glycaemic control **(I HQE)**	Correct risk factors: • Weight reduction and increased physical activity **(I A)** • Smoking cessation **(I C)** • Alcohol control **(IIa C)** • Treat hypertension with lifestyle changes and thiazide diuretic **(I A)** • Statin if CAD **(I A)** • SGLT2 inhibitor in T2DM and CAD **(I A)**
Acute treatment	IV diuretics—either bolus or infusion. Consider higher dose than admission IV nitrates—not for routine use Non-invasive ventilation—consider if there is severe dyspnoea and acidaemia, or if despite treatment the patient has respiratory failure, reduced consciousness or physical exhaustion Invasive ventilation—consider if respiratory failure or reduced consciousness or physical exhaustion despite treatment Inotropes or vasopressors—consider if potentially reversible cardiogenic shock	Treat identifiable precipitating factors or aetiology **(1 C-LD)** IV loop diuretics—improve symptoms and reduce morbidity **(1 B-NR)**; reasonable to use higher dose of loop diuretic or add second diuretic i.e. thiazide or MRA if inadequate decongestion from diuresis **(2a B-NR)** IV nitroglycerin or nitroprusside—consider as adjunct to diuretics for relief of dyspnoea in absence of hypotension **(2b B-NR)** VTE prophylaxis—if not already anticoagulated to prevent venous thromboembolic disease **(1 B-R)**	Diuretics—IV loop diuretics if fluid overload to improve symptoms **(I C)**; consider combination of loop diuretic with thiazide diuretic if resistant oedema not responsive to increase in loop diuretic doses **(IIa B)** Vasodilators—consider if SBP >110 mmHg to improve symptoms and reduce congestion **(IIb B)** Oxygen—if SpO_2_ <90% or PaO_2_ <60 mmHg **(I C)** Ventilatory support—intubation if progressive respiratory failure persisting despite oxygen or non-invasive ventilation **(I C)**; consider non-invasive positive pressure ventilation if respiratory distress (respiratory rate >25 breaths/min, SpO_2_ <90%) **(IIa B)**	IV diuretics—treat pulmonary or peripheral congestion **(I LQE)**; preference for high dose loop diuretic +/− thiazide or MRA Tolvaptan—consider if volume overload and symptomatic hyponatraemia **(II MQE)** IV vasodilators—for relief of dyspnoea if SBP >100 mmHg; nitroglycerin **(II MQE)**, nesiritide **(II HQE)** or nitroprusside **(II VLQE)** Oxygen—if hypoxic, target SpO_2_ >90% **(I MQE)** Non-invasive ventilation—not for routine use **(I MQE)** Inotropes—not for routine use if haemodynamically stable **(I HQE)**	IV loop diuretics—bolus or infusion to improve symptoms of congestion **(I)**; consider adding thiazide or acetazolamide in resistant fluid overload **(IIb)** IV vasodilators—if SBP >110 mmHg for symptomatic relief **(II)** Oxygen—if SpO_2_ <90% or PaO2 <60 mmHg **(I)** Non-invasive ventilation—consider if respiratory rate >25 and SpO_2_ <90% **(IIa)** Intubation—if respiratory failure causing hypoxia, hypercapnia and acidosis which cannot be managed non-invasively **(I)** VTE prophylaxis—if not already anticoagulated **(I)**	Treat precipitating factors or aetiology **(I LQE)** IV loop diuretics—for fluid overload; if resistant consider adding thiazide or MRA **(I LQE)** IV vasodilators—consider to relieve congestive symptoms if SBP >90 mmHg (**II LQE)** Oxygen—if hypoxic, target SpO_2_ <94% **(I VLQE)** Non-invasive ventilation—if remain hypoxic and tachypnoeic despite oxygen therapy **(I HQE)** IV inotropes—consider in peripheral hypoperfusion and congestion refractory to other treatment **(II VLQE)**	Loop diuretics—for fluid retention **(I C)**; if poor response to bolus injections for continuous infusion **(IIa B)** or combination with thiazide or MRA **(IIb C)** Tolvaptan—if poor response to loop diuretic **(IIa A)** or in fluid retention with hyponatraemia **(IIa C)** Vasodilators—treat pulmonary congestion; nitrates **(I B)**, carperitide **(IIa B)** or nicorandil **(IIb C)** Oxygen—if hypoxic, target SpO_2_ >90% **(I C)** Non-invasive ventilation—if dyspnoea and respiratory rate >25 and SpO_2_ <90% **(I A)**
Opiates—not for routine use Ultrafiltration—consider if confirmed diuretic resistance	Cardiogenic shock: • IV inotropes -maintain systemic perfusion **(1 B-NR)** • Temporary MCS—reasonable to support cardiac function if pharmacological methods insufficient **(2a B-NR)**; consider transfer to temporary MCS centre **(2b C-LD)** • PA line placement—consider to define haemodynamics and guide further management **(2b B-NR)**	Vasopressors and/or inotropes—consider if cardiogenic shock unresponsive to standard treatment **(IIb B)** Opiates—routine use not advised unless in selected patients with severe/intractable pain or anxiety **(III C)** Other drugs—thromboembolism prophylaxis (e.g. with low-molecular-weight heparin) if not already anticoagulated and no contraindications (**I A)**	Morphine—not for routine use **(I MQE)**	Inotropes—if SBP <110 mmHg and hypoperfusion not responding to treatment **(IIb)** Vasopressors—consider if poor response to inotropes **(IIb)** Renal replacement therapy—if refractory fluid overload and acute kidney injury **(IIa)** Ultrafiltration—if refractory congestion unresponsive to diuretics **(IIb)**		Tracheal intubation—if remains hypoxaemic (PaO2 <60 mmHg), hypercapnic (PaCO2 >50 mmHg) and acidotic (pH <7.35) despite above measures **(I C)** Cardiogenic shock: • Inotropes/vasopressors—increase cardiac output/maintain systolic blood pressure if persistent peripheral hypoperfusion (**IIa B**) • MCS—consider short-term use **(IIb C)**; transfer to ICU/CCU where MCS available **(I C)** • Swan-Ganz catheterization—determine haemodynamics if unresponsive to appropriate treatment **(I C)** Haemofiltration/haemodialysis/haemodiafiltration—if severe fluid retention refractory to drug therapies **(IIb B)**	
Chronic treatment	Treatment of any causes, comorbidities and precipitating factors Loop diuretics—for fluid retention SGLT2 inhibitor—dapagliflozin or empagliflozin Fluid/salt restriction—only if high levels of intake; requires regular review Multidisciplinary interventions: • Specialist HF multidisciplinary team to deliver care • Education on self-care advice and managing HF • Influenza (annual) and pneumococcal disease (once-only) vaccinations Exercise rehabilitation: • Personalised exercise-based rehabilitation program	Treatment of comorbidities—e.g. hypertension **(1 C-LD)** or AF **(2a C-EO)** and any known aetiology such as amyloidosis **(1 B-NR)** Diuretics—for fluid retention; loop diuretic +/− thiazide diuretic if hypertensive or refractory oedema **(1 B-NR)** SGLT2 inhibitor **(2a B-R)** MRA, ARB or ARNI—consider in select patients, especially if LVEF on lower end of spectrum **(2b B-R)** Dietary sodium restriction—reduce congestive symptoms **(2a C-LD)** Multidisciplinary interventions: • Multidisciplinary team care • Education and support to facilitate HF self-care and exercise **(1 B-R)** • Respiratory illness vaccinations **(2a B-NR)** Exercise rehabilitation: • Exercise training **(1 A)** or cardiac rehabilitation program to improve quality of life **(2a B-NR)**	Treatment for aetiology, CV and non-CV comorbidities—e.g. hypertension, CAD, amyloidosis, AF, valvular heart disease **(I C)** Diuretics—for fluid retention; loop diuretics preferred, although thiazide diuretics may be useful for managing hypertension **(I C)** SGLT2 inhibitor—dapagliflozin or empagliflozin **(I A)** Multidisciplinary interventions: • Multidisciplinary HF management programme **(I A)** • Self-management strategies **(I A)** • Home-based and/or clinic-based programmes **(I A)** • Consider influenza and pneumococcal vaccinations **(IIa B)** Exercise rehabilitation: • Exercise if able **(I A)** • Consider a supervised, exercise-based, cardiac rehabilitation programme in patients with more severe disease, frailty, or with comorbidities **(IIa C)**	Treatment of comorbidities and aetiology—e.g. hypertension and amyloidosis **(I HQE)** Loop diuretics—to manage peripheral oedema **(I MQE)** Candesartan—consider to reduce HF hospitalizations **(II MQE)** MRA—consider if serum potassium <5.0 mmol/L and eGFR >30 mL/min **(II MQE)** Sodium (2-3 g/d) and fluid (max 2 L/d) restriction—consider if fluid retention or congestion not easily controlled with diuretics **(II LQE)** Multidisciplinary interventions: • Referral to HF disease management program if recurrent hospitalizations **(I HQE)** Exercise rehabilitation: • Regular exercise **(I MQE)**	Screen and treat CV and non-CV comorbidities **(I)** Diuretics—for fluid overload **(I)** SGLT2 inhibitors—dapagliflozin or empagliflozin **(I)** ARNI, ARB, beta-blocker, MRA—consider in select patients to reduce risk of HF hospitalization and death **(IIb)** Multidisciplinary interventions: • Enrolment into multidisciplinary team HF management program **(I)** • Self-management strategies **(I)** • Influenza and pneumococcal vaccinations **(IIa)** Exercise rehabilitation: • Regular exercise **(I)** • Cardiac rehabilitation programme **(IIa)**	Treatment of comorbidities and aetiology—e.g. hypertension, T2DM, AF and IHD Diuretics—relieve congestion and improve symptoms; typically loop diuretics but consider thiazides if hypertensive **(I VLQE)** Multidisciplinary interventions: • Multidisciplinary team HF disease management program referral if high-risk **(I HQE)** • Patient-centred education on self-management **(I HQE)** Exercise rehabilitation: • Regular exercise **(I HQE)**	Treatment of comorbidities and aetiology—e.g. hypertension **(I B)** Diuretics—to relieve congestion **(I C)**: • Preference of long-acting loop diuretics e.g. azosemide **(IIb C)** • Tolvaptan—initiate in acute HF and continue following discharge **(IIa C)** ARNI—consider administration **(IIb B)** ACEi/ARB, beta-blocker, MRA—increase dose to maximum tolerable level **(IIb C)** Dietary sodium restriction—consider low-salt diet **(IIa C)** Multidisciplinary interventions: • Comprehensive multidisciplinary team educational programs and medical care **(I C)** • Vaccination of infectious diseases **(IIa C)** Exercise rehabilitation: • Exercise therapy for select patients **(IIa B/C)** • Cardiac rehabilitation programs **(IIa C)**
Advanced treatment	Cardiac transplant—specialist referral if severe refractory symptoms or refractory cardiogenic shock Palliative care—consider referral if worsening HF despite optimal specialist treatment	HF specialty care referral—review management and assess suitability for advanced therapies in patients with advanced HF **(1 C-LD)** Cardiac transplantation—for select patients with advanced HF refractory to guideline-directed medical therapy **(1 C-LD)** Inotropic support—consider for short-term usage as a bridge to transplantation in select patients **(2a B-NR)** or symptom control in palliation **(2b B-NR)** Palliative care—specialist consultation to improve quality of life and alleviate suffering **(1 C-LD)** Fluid restriction—reduce congestive symptoms if hyponatraemia, uncertain benefit **(2b C-LD)**	Heart transplantation -if advanced HF refractory to medical therapy and no absolute contraindications **(I C)** Continuous vasopressors and/or inotropes—consider if low cardiac output and evidence of organ hypoperfusion as bridge to heart transplantation if eligible **(IIb C)** Renal replacement therapy—consider if refractory volume overload and end-stage kidney failure **(IIa C)** Ultrafiltration—consider in refractory volume overload unresponsive to diuretic treatment **(IIb C)**	Specialist centre referral—assessment and management by team with expertise in treating severe HF for patients with acute severe or chronic advanced HF and good life expectancy **(I MQE)** Cardiac transplantation—recommended but specific guidelines for eligibility found elsewhere Inotropes and vasopressors—consider in cardiogenic shock to afford opportunity for evaluation of long-term options **(I MQE)** Palliative care—refer on basis of symptoms rather than life expectancy **(I VLQE)**	Advanced HF team management—review treatment and assess suitability for advanced therapies for advanced HF patients **(I)** Cardiac transplantation—select cases with advanced HF despite optimal medical therapy **(I)** Inotropic support—consider continuous IV inotropes as a bridge to cardiac transplantation if eligible **(IIa)** or continuous/intermittent IV inotropes for symptomatic relief in palliation **(IIb)** Fluid restriction—consider to reduce congestive symptoms if hyponatraemia **(IIb)**	Heart transplant—consider referral for assessment if advanced HF refractory to alternative therapies without overt contraindications **(I LQE)** Palliative care—consider to alleviate symptoms and improve quality of life in advanced HF and early involvement if trajectory towards advanced HF **(I HQE)**	Palliative care—patients with end-stage HF for advance care planning **(I B)**, continued HF treatment to manage complications and symptoms **(I C)** and frequent assessment of physical, mental, and spiritual needs **(II C)**
Surveillance	Review <2 weeks after hospital discharge Every 6 months to check functional status, fluid status, cognition, nutrition, medication and renal function. Monitoring interval should be <2 weeks if clinical status or medication has changed	Review within 7 days of hospital discharge to optimize care and reduce re-hospitalization TTE if unexplained change in clinical status Monitoring: • Consider telemonitoring of PA pressure with implantable haemodynamic monitor **(2b B-R)**	Review 1–2 weeks after hospital discharge to assess signs of congestion and drug tolerance Every 6 months to check symptoms, ECG, BP, full blood count, electrolytes, and renal function. TTE if deterioration in clinical status Monitoring: • Consider non-invasive home telemonitoring **(IIb B)**	Frequency dependent on level of risk: • Lower—every 1 year • Intermediate—every 1–6 months • Higher—1–2 times per month TTE every 1–3 years or after a significant clinical event	Consider referral to primary care for long-term follow-up of stable patients on optimal therapy Monitoring: • Monitoring of pulmonary pressures with implantable device if symptomatic HF and previous admissions **(IIb)**	Review 1–2 weeks after hospital discharge Frequency of regular follow-up should be guided by clinical stability Monitoring: • Telemonitoring or telephone support program if above not available **(I MQE)** • Implantable PA pressure monitoring if persistent symptoms despite optimal care. Requires daily upload and at least weekly review **(II LQE)**	Early review after hospital discharge Every 1 year in HF clinic

ACEi, angiotensin-converting enzyme inhibitor; ACS, acute coronary syndrome; AF, atrial fibrillation; AGREE II, Appraisal of Guidelines Research and Evaluation II; ARB, angiotensin receptor blocker; ARNI, angiotensin receptor/neprilysin inhibitor; BMI, body mass index; BNP, brain natriuretic peptide; BP, blood pressure; CAD, coronary artery disease; CKD, chronic kidney disease; CMR, cardiovascular magnetic resonance; CPET, cardiopulmonary exercise testing; CT, computed tomography; CVD, cardiovascular disease; ECG, electrocardiogram; eGFR, estimated glomerular filtration rate; EI, editorial independence declared; HF, heart failure; IV, intravenous; LA, left atrium; LGE, late gadolinium enhancement; LV, left ventricle; LVEDP, LV end diastolic pressure; LVEF, LV ejection fraction; MCS, mechanical circulatory support; MRA, mineralocorticoid receptor antagonist; NP, natriuretic peptide; NT-proBNP, N-terminal pro-B-type natriuretic peptide; PA, pulmonary artery; PaO_2_, partial pressure of oxygen; PCWP, pulmonary capillary wedge pressure; PET, positron emission tomography; RV, right ventricle; SBP, systolic BP; SCI, statement about conflicts of interest of group members present; SGLT2, sodium-glucose co-transporter-2; SPECT, single-photon emission CT; SpO2, oxygen saturation; T2DM, type two diabetes mellitus; TAPSE, tricuspid annular plane systolic excursion; TOE, transoesophageal echocardiogram; TR, tricuspid regurgitation; TTE, transthoracic echocardiogram; VTE, venous thromboembolism.

ESC and JSC/JHFS:

Level of evidence: A = data derived from multiple randomized clinical trials or meta-analysis. B = data derived from a single randomized trial or non-randomized studies. C = only consensus opinion of experts, and/or small studies, retrospective studies, registries.

Class of recommendation: I, evidence and/or general agreement that the procedure or treatment is beneficial, useful, and effective; Class II: conflicting evidence and/or a divergence of opinion about the usefulness/efficacy of a procedure or treatment; IIa, weight of evidence/opinion is in favour of usefulness/efficacy; IIb, usefulness/efficacy is less well established by evidence/opinion; III, evidence and/or general agreement that the procedure/treatment is not useful/effective and in some cases may be harmful.

AHA/ACC/HFSA:

Level of evidence: A (high quality evidence); B (moderate quality evidence—B-R = randomized study, B-NR = non-randomized); C (C-LD = limited data, C-EO = consensus of expert opinion).

Class of recommendation: Class I = benefit >>> risk; Class IIa = benefit >> risk, Class IIb = benefit ≥ risk; class III = risk ≥ benefit.

SHA:

Class of recommendation: Class I (recommended), usefulness/efficacy is supported by available evidence; Class IIa (should be considered), usefulness/efficacy established by favourable expert opinion on conflicting evidence; Class IIb (may be considered), usefulness/efficacy not well established by evidence or expert opinion; Class III (not recommended), not useful or effective and is potentially harmful based on evidence and/or general agreement.

NHFA/CSANZ and CCS/CHFS:

Level of evidence: High-quality evidence (HQE), confident that true effect is similar to estimated effect; Moderate-quality evidence (MQE), true effect is probably close to estimated effect; Low-quality evidence (LQE), true effect may be markedly different from the estimated effect; very low quality of evidence (VLQE), true effect is probably markedly different from estimated effect.

Class of recommendation: I, strongly recommended—almost all persons would choose intervention; II, weakly recommended—important variation in decision that informed persons are likely to make; III, not recommended.

a—Relationship with industry is reported by any group member.

b—A group member is reported recused when a relevant area is under discussion.

### Areas of agreement

#### Definition of HFpEF

All guidelines defined HFpEF as the presence of clinical signs and symptoms of HF alongside an LVEF ≥50% on cardiac imaging. Six of the documents also required echocardiographic and/or invasive evidence of cardiac abnormalities consistent with LV diastolic dysfunction or raised LV filling pressures,^12,13,15,17,19,21^ and five included raised natriuretic peptides (NPs).^12,13,15,19,21^

#### Diagnostic tests

There was consensus between guidelines that transthoracic echocardiography (TTE) with LV diastolic function assessment and NPs were the key diagnostic tests. If TTE is non-diagnostic, the guidelines recommended that alternative imaging, such as cardiovascular magnetic resonance, should be considered to assess cardiac structure and function or help elucidate aetiology. Cardiopulmonary exercise testing (CPET) should also be considered as per four guidelines to help identify the cause of dyspnoea when uncertain and/or quantify functional capacity.^12,15,17,19^ AHA/ACC/HFSA and JCS/JHFS also recommended CPET in the assessment of eligibility for advanced treatment.

With respect to invasive assessment, four guidelines advised consideration of right heart catheterization to aid with diagnosis, monitor pulmonary artery pressure in select patients, or as a workup for advanced treatment.^12,15,17,19^ Furthermore, four guidelines recommended consideration of endomyocardial biopsy if a specific cause of HF is suspected that would influence therapy, such as myocarditis or amyloidosis.^12,15,17,19^ If an inherited cardiomyopathy is suspected as the underlying aetiology, both AHA/ACC/HFSA and SHA recommended counselling and genetic testing.

#### Prevention

Numerous strategies were recommended primordial for primary and secondary prevention of HFpEF. All guidelines focussed on the control of reversible cardiovascular risk factors through lifestyle modification and pharmacological therapy. Six guidelines strongly recommended tight control of hypertension.^12–15,17,19^ The AHA/ACC/HFSA and CCS/CHFS advised comparatively stricter targets of blood pressure <130/80 mmHg if there is a high risk of cardiovascular disease (CVD), defined as CVD risk >10% by the former. Similarly, five guidelines recommended initiation of SGLT2 inhibitors, typically in patients with type 2 diabetes mellitus (T2DM) and a high risk/presence of CVD.^12,13,15,17,19^ ESC also recommended initiation of SGLT2 inhibitors and finerenone, a non-steroidal mineralocorticoid receptor antagonist (MRA), in T2DM with concomitant chronic kidney disease (CKD).

#### Acute treatment

There was consensus that initiation and/or up-titration of an intravenous loop diuretic was the preferred therapy for relief of acute fluid overload. Should loop diuretic response prove inadequate despite further dose increases or use of a continuous infusion, six guidelines recommended combination with a thiazide,^12–15,17,19^ and four advised consideration of adding an MRA.^12–14,17^ NICE, the only organization to not recommend a thiazide, suggested consideration, but that further evidence is required.

If pulmonary congestive symptoms are refractory to standard treatment, and in the absence of symptomatic hypotension, then intravenous vasodilators such as sodium nitroprusside can be considered as per six guidelines to optimise preload and afterload.^12–15,17,19^ Selected guidelines highlighted concomitant myocardial ischaemia, mitral regurgitation, or severe hypertension as comorbidities where vasodilators may prove more effective. Despite the concordance in indication, there was minor disparity in the systolic blood pressure threshold for withholding vasodilators, varying between <90 and 110 mmHg. Extra care should also be taken in patients with valvular stenosis.

Five guidelines provided a class 1 recommendation for the use of oxygen therapy in the presence of hypoxia.^13–15,17,19^ In the event of persistent hypoxia and tachypnoea despite oxygen therapy, five guidelines also recommended consideration of non-invasive ventilatory support,^13,15,17,19,22^ and four recommended invasive ventilation if there was progressive respiratory failure and acidosis despite the aforementioned treatment.^15,17,19,22^

Upon progression to potentially reversible cardiogenic shock, consideration of inotropes was advised by six guidelines.^12,13,15,17,19,22^ Vasopressors were recommended as a possible adjunct by four guidelines,^15,17,19,22^ although both ESC and NHFA/CSANZ warned of the potential for increased afterload and further reduction in end-organ perfusion. Crucially, routine use of these therapies outside of cardiogenic shock was discouraged given their extensive side effect profiles.

#### Chronic treatment

There was unanimity amongst the guidelines that diuretics should be given to treat fluid retention in chronic HFpEF. All guidelines reported an initial preference for loop diuretics, aside from SHA, which did not specify. All guidelines also recommended the identification and treatment of any known aetiology and comorbidities associated with HFpEF to improve outcomes. Common cardiovascular and non-cardiovascular comorbidities acknowledged across the guidelines included atrial fibrillation (AF), valvular heart disease, hypertension, diabetes mellitus, anaemia, sleep apnoea, and renal dysfunction. Four guidelines (published after 2021) recommended SGLT2 inhibitors as a pharmacological disease-modifying therapy for HFpEF^12,15,19,22^; these were updated following publication of positive results from the DELIVER^9^ and EMPEROR-PRESERVED^10^ trials.

All guidelines were in agreement that a multidisciplinary HF programme should be offered. Patient education on HF self-management was also a common theme, and five guidelines recommended respiratory illness vaccinations.^12,15,17,19,21^ All guidelines recommended exercise therapy, of which five specified cardiac rehabilitation programs.^12,15,17,19,21^

#### Advanced treatment

Advanced HF is primarily defined by the persistence of severe symptoms despite maximal medical therapy. Recommendations provided by the guidelines were not specific to advanced HF secondary to HFpEF, and the vast majority will have reduced LVEF. Nevertheless, management of these patients under an advanced HF specialist team was advised. Six guidelines recommended consideration of cardiac transplantation as the gold standard for treatment,^12–15,19,21^ recognizing that advanced comorbidity in HF excludes a high proportion of patients from candidacy.^23^ Five guidelines agreed that palliative care input was beneficial in alleviating symptoms and improving quality of life.^12–14,17,21^ Early palliative intervention, intensifying with disease progression, alongside supportive measures for symptomatic relief were key in holistically addressing patient needs.

### Areas of disagreement

#### Imaging parameters and biomarkers

TTE was the preferred first-line imaging modality for diagnosis of LV diastolic dysfunction and structural heart disease. Although many parameters and their diagnostic thresholds were similar, noteworthy exceptions included ESC defining an early transmitral filling velocity (E) to early myocardial relaxation velocity (e’) ratio (E/e’) at rest >9 and AHA/ACC/HFSA defining a left atrial volume indexed to body surface area (LAVI) ≥29 mL/m^2^; both of these thresholds were lower than those provided by other guidelines.

Differences were also observed in the interpretation of NP values; some guidelines used distinct diagnostic thresholds based on the acuity of HF, whereas others considered the effect of AF, CKD, and age on NP cut-offs and adjusted accordingly. Moreover, NICE and NHFA/CSANZ were notable for recommending a comparatively higher diagnostic threshold of N-terminal pro-B-type NP (NT-proBNP) at >400 ng/L and >450 (age <50 years), >900 (age 50–75 years) or >1800 ng/L (age >75 years), respectively.

#### Screening and diagnostic algorithm

Widespread support for one specific diagnostic algorithm was lacking. While ESC, AHA/ACC/HFSA, JCS/JHFS and SHA discussed the use of score-based diagnostic algorithms, implementation and choice of score was typically left to physician discretion. When diagnostic score or biomarkers and resting TTE are equivocal, diastolic stress testing was only recommended by ESC and SHA. The former discussed the benefits of invasive haemodynamic exercise testing as a confirmatory test, while the latter recommended assessment of diastolic function when stressed via CPET and TTE. The AHA/ACC/HFSA and JCS/JHFS advised diastolic stress testing when diagnosis is uncertain by offering exercise TTE in the main text, but did not provide a formal recommendation.

With regards to screening patients at risk of developing HF, only the AHA/ACC/HFSA recommended usage of NPs and risk scores to prevent onset. The challenges were acknowledged, especially given the heterogeneous aetiology and risk factors underlying HF.

#### Acute treatment

The use of tolvaptan in an acute setting was only recommended in the CCS/CHFS and JCS/JHFS guidelines. Both encouraged consideration in the presence of fluid overload and hyponatraemia, although JCS/JHFS also stipulated oedema refractory to loop diuretics as a second indication. The AHA/ACC/HFSA and NHFA/CSANZ guidelines also suggested consideration, but not as a formal recommendation given the paucity of positive results from trials to date. Similarly, adjunctive acetazolamide was recommended only by the SHA in patients with resistant oedema or insufficient symptomatic response to loop diuretics.

Ultrafiltration, as a therapeutic ‘last resort’ for refractory fluid overload, was recommended by the JCS/JHFS, NICE, and SHA guidelines. The ESC and NHFA/CSANZ guidelines discussed the possible benefits of initiating ultrafiltration, but did not recommend its routine use.

#### Chronic treatment

The role of combination diuretic strategies in chronic HFpEF was inconsistent between guidelines. The AHA/ACC/HFSA, ESC, and NHFA/CSANZ guidelines recommended consideration of thiazides as an adjunct if the patient is hypertensive. Additionally, oedema refractory to loop diuretics irrespective of blood pressure was a second indication only identified in the AHA/ACC/HFSA guideline. The JCS/JHFS guideline was unique in its preference for long-acting loop diuretics such as azosemide over furosemide, alongside the long-term continuation of tolvaptan if initiated in an acute setting. Four guidelines recommended dietary sodium restriction to reduce congestive symptoms.^12,14,17,21^ However, the paucity of evidence regarding efficacy and level of restriction was acknowledged, and there remains little consensus on whether this is an effective treatment strategy in chronic HFpEF.

There is inconsistent guidance on the benefit of renin-angiotensin-aldosterone system (RAAS) inhibitors and beta-blockers for treating chronic HFpEF. Although generally acknowledged to have weak evidence, four guidelines recommended treatment with an MRA and angiotensin receptor blocker (ARB) (class II–IIb recommendation).^16,19,21,23^ Alternatively, instead of an ARB, three guidelines suggested consideration of an angiotensin-receptor/neprilysin inhibitors (ARNI)^16,21,23^ and only the JCS/JHFS guideline suggested an angiotensin-converting enzyme inhibitors (ACEi). Two guidelines suggested the use of a beta-blocker.^21,23^ The AHA/ACC/HFSA guideline indicated stronger support for MRA, ARB, and ARNI initiation in those with an LVEF closer to 50%.

#### Surveillance

There were inconsistent recommendations across the guidelines for clinical surveillance frequency and constitution, with often only generic statements provided. For stable patients with chronic HFpEF, both ESC and NICE recommended six-monthly reviews whereas both JCS/JHFS and CCS/CHFS advised at least annual surveillance, with higher risk patients warranting more frequent follow-up for the latter. No other guidelines provided information on surveillance frequency for patients with chronic HFpEF. Following hospital discharge for initial admission with acute HF, the ESC, NICE, and NHFA/CSANZ guidelines recommended review within 2 weeks, while AHA/ACC/HFSA recommended review within 1 week. The remaining guidelines did not specify a timeframe for review after hospital discharge. AHA/ACC/HFSA, SHA and NHFA/CSANZ weakly recommended monitoring of pulmonary artery pressure with an implantable device in symptomatic patients (class II–IIb recommendation). ESC and NHFA/CSANZ also considered non-invasive telemonitoring, where patients are typically required to self-record measurements at home.

## Discussion

We identified ten clinical practice guidelines and recommendations, of which seven were rigorously developed, on the diagnosis and management of patients with HFpEF. There is consensus on the definition of HFpEF and steps to diagnose the syndrome with the role of biomarkers, non-invasive imaging and invasive tests. All guidelines addressed the pharmacological management of chronic HFpEF, with an emphasis on the role of diuretics and vasodilators for symptom control. Recently published guidelines advocate the use of SGLT2 inhibitors to improve the quality of life and reduce HF hospitalizations. Cardiac transplantation can be considered in advanced HFpEF, for those eligible. The very clear role to prevent the development of HFpEF by addressing cardiometabolic comorbidities is key. Multidisciplinary team (MDT) involvement throughout was key in ensuring personalized care.

**Figure 2:**
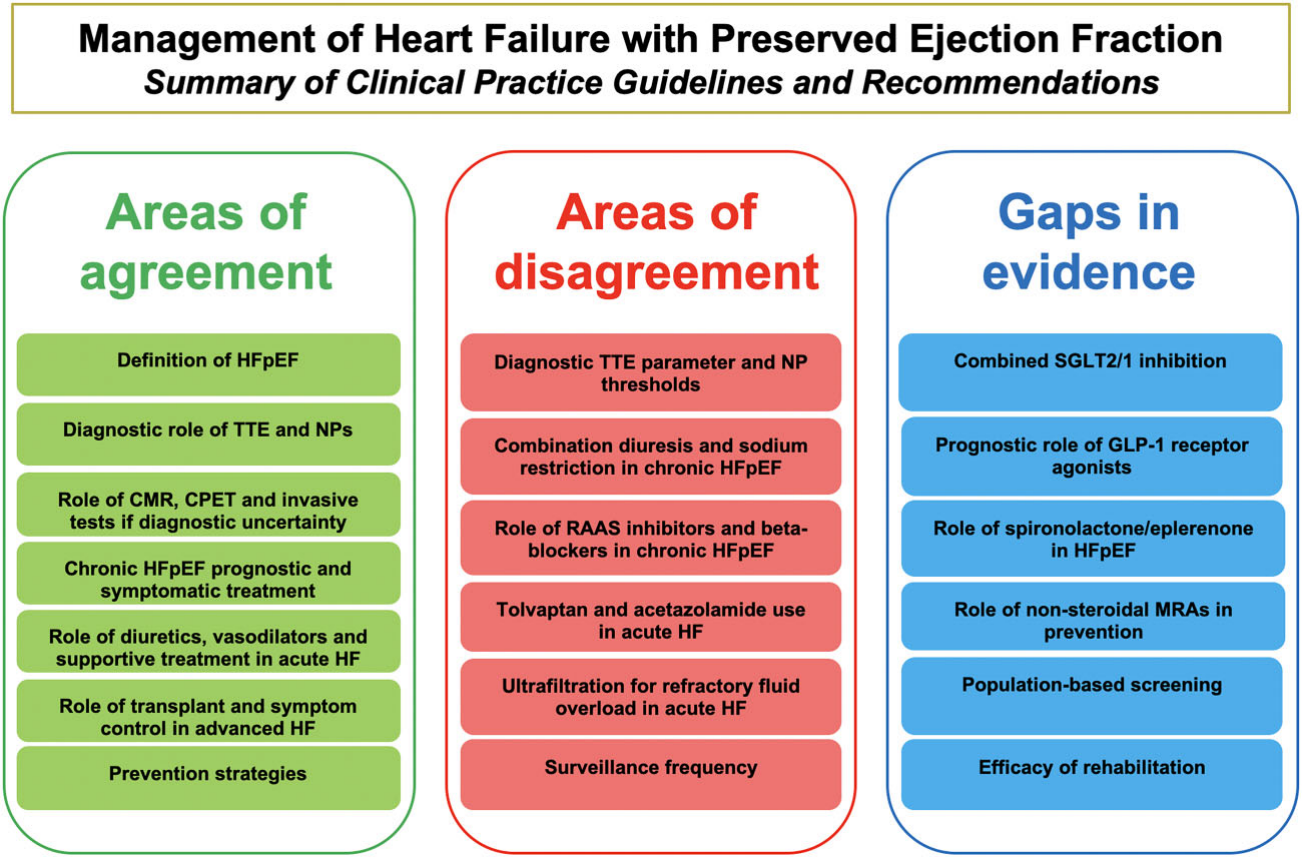
Summary of clinical practice guidelines and recommendations on heart failure with preserved ejection fraction management. CMR, cardiovascular magnetic resonance; CPET, cardiopulmonary exercise testing; GLP-1, glucagon-like peptide-1; HFpEF, heart failure with preserved ejection fraction; MRA, mineralocorticoid receptor antagonist; NP, natriuretic peptide; RAAS, renin-angiotensin-aldosterone system; SGLT2/1, sodium-glucose co-transporter 2/1; TTE, transthoracic echocardiography.

Discrepancies are present in the thresholds of key biomarker and imaging parameters used to diagnose HFpEF, the specific treatment strategies used to manage acute and chronic HFpEF (beyond SGLT2 inhibitors and diuretics), and the frequency of surveillance. There was limited guidance on screening for HFpEF in the general population, use of diagnostic scoring systems, and the role of newly emerging therapies. *Figure 2* provides a summary of the areas of agreement, disagreement and potential gaps in evidence requiring future clarification.

### Imaging parameters and biomarkers

Guidance on the use of TTE for diagnosis of LV diastolic dysfunction in HFpEF largely stems from the 2016 American Society of Echocardiography/European Association of Cardiovascular Imaging guidelines.^24^ Key parameters were e’, average E/e’ ratio, LAVI and tricuspid regurgitation peak velocity. Diagnostic thresholds for these varied occasionally. ESC derived recommended cut-offs from data analysing pivot points associated with mortality risk, and consequently settled on E/e’ >9 as suggestive of diastolic dysfunction.^25^ It should be noted that there was an acceleration of risk above 14, and indeed, E/e’ >14 was indicative of diastolic dysfunction in other guidelines. Left atrial strain, a comparatively novel marker, has been shown to accurately categorise diastolic dysfunction and correlate with its progression.^26,27^ It has already been touted as a potential new parameter in the British Society of Echocardiography's upcoming diastolic dysfunction guideline, but did not feature in the guidelines reviewed to date.

The thresholds for NPs and their use in the diagnosis of HFpEF remain discordant. Although elevated levels correlate with worsening diastolic dysfunction, NPs are lower in HFpEF compared with HFrEF,^28^ and can be in the normal range for some patients.^29^ Acknowledgement that levels can also rise with age led to NHFA/CSANZ advising differing age-related rule-in thresholds, including a noticeably elevated threshold for patients >75 years (NT-proBNP >1800 ng/L).^30^ Comorbidities such as AF and obesity, which are common in HFpEF, also affect NPs and the ESC guideline suggested a raised threshold in those with AF and proposed lowering in obesity.^31^ Nevertheless, challenges associated with NPs extend beyond their initial diagnostic utility. Treatment of comorbidities may reduce levels without necessarily indicating improvement in HF, potentially confounding the interpretation of serial NP measurements.^32^ This partially explains the difficulty in utilizing NPs in prognosis, risk stratification, and guidance of management.

### Score-based diagnostic algorithms

Two scores, the H_2_PEF and HFA-PEFF, have been developed to aid diagnosis of HFpEF, especially when initial investigations are equivocal despite high clinical suspicion.^33,34^ The ESC proposes HFA-PEFF be utilized provided the necessary expertise is available, while the AHA/ACC/HFSA does not state a preference between the two. Both scores assess the likelihood of diagnosis, although in a significant proportion diagnostic uncertainty remains and further investigations, such as diastolic stress testing, are required. It should be noted that discrepancy between the scores exist, and studies assessing accuracy have returned mixed results.^35,36^

### Diuretics

Use of diuretics for symptomatic relief of fluid retention in chronic HFpEF is well-established. Loop diuretics, conventionally furosemide, are the drugs of choice and the lowest effective dose should be given in the first instance. Yet, there is no evidence that diuretics improve HFpEF prognosis, and there is some evidence to suggest they may stimulate the sympathetic nervous system and RAAS.^37,38^ Indeed, it is this very reason which led to the JCS/JHFS guideline being an outlier in recommending the long-acting azosemide over furosemide, given the former's comparatively reduced neurohormonal impact.^37^

Loop diuretics are the backbone of acute fluid overload management, and when initially ineffective there is preference for initial dose up-titration over combination diuresis strategies. However, in the event of refractory oedema, addition of a thiazide or thiazide-like diuretic such as metolazone can be considered to maximize diuretic synergy through sequential nephron blockade.^39^ Close monitoring of renal function and electrolytes is crucial, given the increased propensity for derangement.^40^ Adjunctive tolvaptan was another option provided by the CCS/CHFS and JCS/JHFS guidelines. The literature remains ambiguous, and while some symptomatic improvement has been reported, no effect on mortality or rehospitalization has yet been observed.^41–44^ Alternatively, addition of acetazolamide alongside loop diuretics has recently been shown to increase incidence of successful decongestion across the LVEF spectrum.^45^ Although the ESC in their 2023 update emphasised the need for further data prior to advocating usage, the SHA have recommended consideration.

### SGLT2 and SGLT1 inhibition

The identification of SGLT2 inhibitors as the first disease-modifying therapy for HFpEF has led to early adoption in all recent guideline updates. Trials met their endpoints largely through a reduction in HF hospitalizations,^8,9^ and produced similar results when LVEF was between 25% and 65%.^46^ The positive effects of empagliflozin were attenuated when LVEF was >65%, unlike dapagliflozin where reduction in the primary endpoint persisted across the spectrum of LVEF.^46,47^ Nevertheless, a pooled data meta-analysis later identified significant reduction in HF hospitalizations and cardiovascular death for SGLT2 inhibitors across the LVEF spectrum.^48^

The SOLOIST-WHF^49^ trial investigated combined SGLT2 and SGLT1 inhibition via sotagliflozin in recently worsening HFpEF with T2DM, although the trial was terminated early following the COVID-19 pandemic and insufficient funding. Positive results have led to the ESC and AHA/ACC/HFSA recommending sotagliflozin in certain populations, but the benefits of widespread use remain unclear. Adverse events, such as diarrhoea, genital infections and diabetic ketoacidosis, have also been more commonly associated with sotagliflozin compared with placebo.^50^ The ongoing SOTA-P-CARDIA (NCT05562063) trial aims to further delineate potential benefits of sotagliflozin in HFpEF without T2DM. Studies comparing combined SGLT2 and SGLT1 inhibition with selective SGLT2 inhibition are also required to distinguish the effect of SGLT1 inhibitory action.

### GLP-1 receptor agonists

As yet, there are no recommendations in the guidelines regarding the use of GLP-1 receptor agonists in HFpEF. The recently published STEP-HFpEF^51^ and STEP-HFpEF DM^52^ trials concluded that semaglutide treatment resulted in fewer symptoms, improved exercise function and increased weight loss compared with placebo. These effects were consistent across patient demographics and clinical characteristics.^53^ However, the effect of semaglutide on HF hospitalization or mortality was only investigated as a composite secondary endpoint. Furthermore, these trials predate the advent of SGLT2 inhibitors as key therapeutics, and the effects of combination therapy remains unknown. The SUMMIT trial (NCT04847557) is currently investigating the effect of tirzepatide, a combined glucose-dependent insulinotropic polypeptide and GLP-1 receptor agonist, on HF symptoms, cardiovascular death and HF events. Given the promising results observed thus far, a notable increase in research is anticipated.

### Role of RAAS inhibitors and beta-blockers

Treatment of concurrent comorbidities remains the cornerstone of HFpEF management, and many patients will already be taking a combination of ACEi, ARB, ARNI, MRA, or beta-blocker for other indications. In part, this is due to the change in the LVEF definition of HFpEF in recent guidelines, with the pillars of prognostically important HFrEF therapy extrapolated to HFmrEF (LVEF <50%).^54^

Initiation of an MRA or ARB for HFpEF was appropriate as per four guidelines, while three recommended an ARNI. Despite major trials demonstrating no significant improvement in primary outcomes for MRAs, ARBs or ARNIs, post-hoc analyses and secondary outcome data suggest some potential benefit, especially in those with an LVEF closer to 50%.^55–60^ The ambiguity of the evidence, and the uncertainty surrounding the ideal target population of these medications, explains the weak recommendations provided, although the AHA/ACC/HFSA suggest stronger consideration in those with comparatively lower LVEF. The SPIRIT-HF (NCT04727073) and SPIRRIT-HFpEF (NCT02901184) trials are ongoing and will provide further insight into the role of MRAs in HFpEF.

Given only the recent publication of large clinical trials, the ESC was alone in recommending the non-steroidal MRA, finerenone, to reduce HF hospitalizations in patients with T2DM and CKD.^61,62^ Broadening the scope of those eligible for therapy remains a possibility, and is being investigated in the Moonraker clinical development program which encompasses a series of HF trials for finerenone. The first of these trials, FINEARTS-HF (NCT04435626), is expected to be published soon.

### Multidisciplinary interventions

Involvement of the wider MDT is crucial in providing personalized treatment and effective preventive care. Cardiac rehabilitation typically consists of medical assessment, patient education on lifestyle modifications, psychosocial support and exercise training.^63^ There is a wealth of evidence for its efficacy in HFrEF through improvement in quality of life and reduction in hospitalization, but significantly less data exists in HFpEF.^63,64^ Five guidelines still recommended cardiac rehabilitation, but the need for further research is acknowledged. The REHAB-HFpEF (NCT05525663) and REACH-HFpEF trials are underway and aim to investigate the effect of rehabilitation on HFpEF outcomes.

Cardiac rehabilitation constitutes an important aspect of HFpEF management, and the objectives often overlap with nurse-led programmes, self-management strategies, and palliative care involvement.^63^ The majority of guidelines emphasized the importance of addressing palliative care needs by the wider MDT. This should start early in disease trajectory, with referral to specialist palliative care clinicians if patient needs are otherwise being unmet. This highly personalized approach and unpredictable disease progression can lead to uncertainty over timing of referral, to the detriment of the patient’s quality of life.^65^

The benefits of salt restriction in HFpEF remain unclear, and appear to vary with level of restriction, target population and involvement of the wider MDT.^66^ The SODIUM-HF trial and a subsequent meta-analysis have recently reported no reduction in HF clinical events with sodium restriction, although symptomatic improvement remains a possibility.^66,67^ Ensuring compliance with specific diets has proved challenging in studies, and strategies such as delivering pre-prepared meals and nurse-led education have been trialled.^68,69^ Given the lack of certainty, further research is warranted to understand if sodium restriction is beneficial.

### Surveillance

Differences in the recommended monitoring frequency for stable HFpEF were evident, with two guidelines advocating for minimum 6 monthly^15,21^ and annual reviews.^14,17^ Surveillance frequency should increase with worsening clinical stability, yet there remains no consensus on appropriate level of monitoring. Studies investigating optimum monitoring intervals from an outcome and cost-effectiveness perspective are lacking. Consequent disparity in clinical practice may lead to superfluous or insufficient monitoring of patients. Consensus is also lacking on the constitution of monitoring, and whether HF specialists, nurses, or primary care physicians should be responsible for routine follow-up.

The increasing uptake of non-invasive home telemonitoring systems, as recommended by the ESC and NHFA/CSANZ guidelines, further complicates the above. They can be used in optimization of treatment or detection of deterioration, although the ESC favours the former.^15^ Benefits include increasing accessibility for patients with poor mobility and who are in geographically underserved areas, and supplementing the paucity of HF-specific physicians and nurses across many countries.^70^ A Cochrane systematic review observed that home telemonitoring reduced mortality and HF hospitalizations, although neutral trials subsequently published may attenuate these results.^71,72^

### Screening

The AHA/ACC/HFSA discuss the utility of NPs in screening for HF in at-risk patients. Two clinical trials observed reduced rates of adverse cardiovascular outcomes, and achieved their primary endpoints following NP screening (BNP >50 pg/L and NT-proBNP >125 pg/L) and subsequent management.^73,74^ Importantly, the number of clinical events in these studies were low, and the effect that screening had on HFpEF specifically is unknown. Further evidence is required, especially given a proportion of patients with HFpEF have normal NP levels, to avoid unnecessary investigations where thresholds are too low to ensure cost-effectiveness and avoid undue patient anxiety.

### Limitations

Some limitations require attention given the potential for bias. First, our systematic review was restricted to guidelines published in the English language. We mitigated the impact of this on our results by following a robust systematic methodology in accordance with PRISMA guidelines. Second, detailed recommendations on the management of comorbidities in HFpEF were not scrutinized and are beyond the scope of this review. Lastly, we did not assess the validity of individual recommendations made by the guidelines. Instead, we ensured all included studies were rigorously developed using AGREE II scoring.

## Conclusions

There is concordance amongst the guidelines for management of HFpEF on the use of diuretics for symptomatic relief, SGLT2 inhibitors to provide prognostic benefit, and involvement of the wider MDT to provide holistic care. However, the absence of a standardized diagnostic algorithm was evident, stemming from varying thresholds for NP and TTE parameters, and diverse utilization of diagnostic scores. Increased consensus on the diagnosis of HFpEF may aid the application of therapies across geographical boundaries. Further research is justified to investigate the potential benefits of combined SGLT2 and SGLT1 inhibition, GLP-1 receptor agonists, non-steroidal MRAs, and cardiac rehabilitation in HFpEF management.

## Supplementary Material

qcae053_Supplemental_File

## Data Availability

The data underlying this article are available in the article and in its online supplementary material. Any further data that readers would like, can be made available on reasonable request to the corresponding author.
