# Supplementary material for: Heart failure with preserved ejection fraction management: a systematic review of clinical practice guidelines and recommendations
Source: Eur Heart J Qual Care Clin Outcomes. 2024 Jun 25;10(7):571–89. doi: 10.1093/ehjqcco/qcae053 (PMC11537231; doi:10.1093/ehjqcco/qcae053)
Supplement: qcae053_Supplemental_File [file qcae053_supplemental_file.docx]

**Supplementary material online**

**Search syntax**

Ovid MEDLINE(R) ALL <1946 to February 27, 2024>

1 "heart failure with preserved ejection fraction".ab,ti. 5077

2 ("Heart failure" and "preserved ejection fraction").ab,ti. 7351

3 "diastolic dysfunction".ab,ti. 11744

4 HFpEF.ab,ti. 5120

5 Heart Failure, Diastolic/ 865

6 "heart failure with normal ejection fraction".ab,ti. 128

7 ("heart failure" and "normal ejection fraction").ab,ti. 476

8 "diastolic heart failure".ab,ti. 1361

9 1 or 2 or 3 or 4 or 5 or 6 or 7 or 8 20136

10 (guideline* or "practice guideline" or "consensus development conference" or "consensus development conference, NIH" or guidance* or "position paper" or "position stand" or statement* or recommendation* or consensus or "practice parameter*").pt. 48115

11 (guideline* or standard* or recommendat* or "practice parameter*" or "position statement*" or "policy statement*" or CPG* or "best practice*" or guidance* or "position paper" or "position stand" or recommendation* or consensus).ti. 326239

12 (care adj2 (path or paths or pathway or pathways or map or maps or plan or plans or standard)).ti. 9240

13 ((critical or clinical or practice) adj2 (path or paths or pathway or pathways or protocol*)).ti. 4924

14 (guideline* or standards or consensus* or recommendat*).au. 9

15 exp Guideline/ 38199

16 10 or 11 or 12 or 13 or 14 or 15 351953

17 9 and 16 247

18 limit 9 to (consensus development conference or consensus development conference, nih or government publication or guideline or legislation or practice guideline) 18

19 17 or 18 247

20 limit 19 to yr="2013 -Current" 207

Embase <1974 to 2024 February 27>

1 "heart failure with preserved ejection fraction".ab,ti. 9263

2 ("Heart failure" and "preserved ejection fraction").ab,ti. 13839

3 "diastolic dysfunction".ab,ti. 24191

4 HFpEF.ab,ti. 11314

5 heart failure with preserved ejection fraction/ 13985

6 "heart failure with normal ejection fraction".ab,ti. 253

7 ("heart failure" and "normal ejection fraction").ab,ti. 1056

8 "diastolic heart failure".ab,ti. 2797

9 1 or 2 or 3 or 4 or 5 or 6 or 7 or 8 44087

10 (guideline* or standard* or recommendat* or "practice parameter*" or "position statement*" or "policy statement*" or CPG* or "best practice*" or guidance* or "position paper" or "position stand" or recommendation* or consensus).ti. 415698

11 (care adj2 (path or paths or pathway or pathways or map or maps or plan or plans or standard)).ti. 13598

12 ((critical or clinical or practice) adj2 (path or paths or pathway or pathways or protocol*)).ti. 6658

13 (guideline* or standards or consensus* or recommendat*).au. 27

14 exp practice guideline/ 737875

15 10 or 11 or 12 or 13 or 14 1024016

16 9 and 15 2336

17 limit 16 to yr="2013 -Current" 2141

| **Appendix Table 1.**  **Website searches of guideline development organizations, including websites**   \| **Organization Responsible for Guideline Development** \| **Country** \| **Website Searched** \| \| --- \| --- \| --- \| \| American Academy of Family Physicians \| United States \| www.aafp.org \| \| American Association of Thoracic Surgery \| United States \| www.aats.org \| \| American College of Cardiology \| United States \| www.acc.org \| \| American College of Physicians \| United States \| www.acponline.org \| \| American College of Surgeons \| United States \| www.facs.org \| \| American College for Preventive Medicine \| United States \| www.acpm.org \| \| American College of Radiology \| United States \| www.acr.org \| \| American Geriatrics Society \| United States \| www.americangeriatrics.org \| \| American Heart Association \| United States \| www.americanheart.org \| \| American Society of Echocardiography \| United States \| www.asecho.org \| \| American Society of Nuclear Cardiology \| United States \| www.asnc.org \| \| American Medical Association \| United States \| www.ama-assn.org \| \| Australian Medical Association \| Australia \| www.ama.com.au \| \| British Cardiovascular Society \| United Kingdom \| www.bcs.com \| \| British Society of Echocardiography \| United Kingdom \| www.bsecho.org \| \| British Society for Heart Failure \| United Kingdom \| www.bsh.org.uk \| \| Canadian Cardiovascular Society \| Canada \| www.ccs.ca \| \| Canadian Heart Failure Society \| Canada \| www.heartfailure.ca \| \| Canadian Task Force on Preventive Health Care \| Canada \| www.canadiantaskforce.ca \| \| Cardiac Society of Australia and New Zealand \| Australia \| www.csanz.edu.au \| \| Centers for Disease Control and Prevention/American Heart Association \| United States \| www.cdc.gov \| \| Department of Health \| United Kingdom \| www.dh.gov.uk/en \| \| European Association of Cardiovascular Imaging \| Europe \| www.escardio.org/Sub-specialty-communities/European-Association-of-Cardiovascular-Imaging-(EACVI) \| \| European Association for Cardio-Thoracic Surgery \| Europe \| www.eacts.org \| \| European Society of Cardiology \| Europe \| www.escardio.org \| \| Heart Failure Association of the ESC \| Europe \| www.escardio.org/Sub-specialty-communities/Heart-Failure-Association-of-the-ESC-(HFA) \| \| Heart Failure Society of America \| United States \| www.hfsa.org \| \| Heart Rhythm Society \| United States \| www.hrsonline.org \| \| Heart Valve Society \| United States \| www.heartvalvesociety.org \| \| Japanese Circulation Society \| Japan \| www.j-circ.or.jp \| \| Japanese Heart Failure Society \| Japan \| www.asas.or.jp/jhfs \| \| Japanese Society of Echocardiography \| Japan \| www.jse.gr.jp \| \| Japanese Society of Ultrasonic in Medicine \| Japan \| www.jsum.or.jp \| \| National Health and Medical Research Council \| Australia \| www.nhmrc.gov.au \| \| National Heart Foundation \| Australia \| www.heartfoundation.org.au \| \| National Heart Lung and Blood Institute \| United States \| www.nhlbi.nih.gov/guidelines \| \| National Institute for Health and Care Excellence \| United Kingdom \| www.nice.org.uk \| \| New Zealand Guidelines Group \| New Zealand \| www.nzgg.org.nz \| \| Royal College of General Practitioners \| United Kingdom \| www.rcgp.org.uk \| \| Scottish Intercollegiate Guidelines Network \| United Kingdom \| www.sign.ac.uk \| \| Society of Cardiovascular Computed Tomography \| United States \| www.scct.org \| \| Society for Cardiovascular Magnetic Resonance \| United States \| www.scmr.org \| \| Society of Critical Care Medicine \| United States \| www.sccm.org \| \| The Society for Cardiovascular Angiography and Interventions \| United States \| www.SCAI.org \| \| The Society of Thoracic Surgeons \| United States \| www.sts.org \| \| U.S. Preventive Services Task Force \| United States \| www.ahrq.gov \| \| World Heart Federation \| International \| www.world-heart-federation.org \| \| World Health Organization \| International \| www.who.int \| |
| --- | --- | --- | --- | --- | --- | --- | --- | --- | --- | --- | --- | --- | --- | --- | --- | --- | --- | --- | --- | --- | --- | --- | --- | --- | --- | --- | --- | --- | --- | --- | --- | --- | --- | --- | --- | --- | --- | --- | --- | --- | --- | --- | --- | --- | --- | --- | --- | --- | --- | --- | --- | --- | --- | --- | --- | --- | --- | --- | --- | --- | --- | --- | --- | --- | --- | --- | --- | --- | --- | --- | --- | --- | --- | --- | --- | --- | --- | --- | --- | --- | --- | --- | --- | --- | --- | --- | --- | --- | --- | --- | --- | --- | --- | --- | --- | --- | --- | --- | --- | --- | --- | --- | --- | --- | --- | --- | --- | --- | --- | --- | --- | --- | --- | --- | --- | --- | --- | --- | --- | --- | --- | --- | --- | --- | --- | --- | --- | --- | --- | --- | --- | --- | --- | --- | --- | --- | --- | --- | --- | --- | --- | --- | --- | --- | --- | --- | --- |

**Appendix Table 2.**

**AGREE II instrument rigour of development domain results**

| Guideline | Reviewer | Method to search for evidence | Criteria to select evidence | | Strengths and limitations of evidence | | Methods for formulating recommendations | Health benefits, side effects, and risks | Link between recommendations and evidence | Procedures for external expert review | Updating process | Domain score, % |
| --- | --- | --- | --- | --- | --- | --- | --- | --- | --- | --- | --- | --- |
|  |  |  | |  | |  |  |  |  |  |  |  |
| NICE | A | 7 | | 7 | | 6 | 7 | 6 | 7 | 3 | 5 | 88% |
|  | B | 7 | | 7 | | 7 | 7 | 7 | 7 | 4 | 5 |  |
| AHA/ACC/HFSA | A | 6 | | 6 | | 5 | 7 | 7 | 7 | 7 | 6 | 85% |
|  | B | 6 | | 3 | | 5 | 5 | 7 | 7 | 6 | 6 |  |
| ESC | A | 4 | | 3 | | 6 | 6 | 7 | 7 | 6 | 5 | 72% |
|  | B | 4 | | 2 | | 4 | 3 | 6 | 7 | 5 | 6 |  |
| CCS/CHFS | A | 5 | | 5 | | 5 | 6 | 7 | 7 | 6 | 2 | 69% |
|  | B | 4 | | 3 | | 2 | 4 | 7 | 7 | 5 | 3 |  |
| SHA | A | 5 | | 5 | | 5 | 6 | 6 | 6 | 6 | 2 | 68% |
|  | B | 5 | | 3 | | 4 | 5 | 6 | 6 | 5 | 2 |  |
| NHFA/CSANZ | A | 5 | | 3 | | 5 | 6 | 6 | 6 | 6 | 1 | 66% |
|  | B | 4 | | 2 | | 4 | 5 | 7 | 7 | 6 | 1 |  |
| JCS/JHFS | A | 2 | | 2 | | 5 | 3 | 6 | 6 | 3 | 2 | 55% |
|  | B | 2 | | 1 | | 5 | 5 | 6 | 7 | 5 | 2 |  |
| KSHF | A | 1 | | 2 | | 2 | 1 | 6 | 6 | 2 | 1 | 43% |
|  | B | 2 | | 2 | | 3 | 3 | 7 | 6 | 3 | 2 |  |
| TSC | A | 1 | | 1 | | 2 | 4 | 5 | 4 | 2 | 1 | 35% |
|  | B | 1 | | 1 | | 3 | 1 | 5 | 5 | 2 | 2 |  |
| HFCT | A | 1 | | 2 | | 3 | 2 | 4 | 5 | 2 | 1 | 32% |
|  | B | 1 | | 2 | | 2 | 2 | 2 | 3 | 2 | 2 |  |

**PRISMA Reporting Checklist**

| **Section/topic** | **#** | | **Checklist item** | | **Reported on page #** |  |
| --- | --- | --- | --- | --- | --- | --- |
| **TITLE** | | | | |  |  |
| Title | 1 | | Identify the report as a systematic review, meta-analysis, or both. | | Title page 1 |  |
| **ABSTRACT** | | | | |  |  |
| Structured summary | 2 | | Provide a structured summary including, as applicable: background; objectives; data sources; study eligibility criteria, participants, and interventions; study appraisal and synthesis methods; results; limitations; conclusions and implications of key findings; systematic review registration number. | | Abstract page 3 |  |
| **INTRODUCTION** | | | | |  |  |
| Rationale | 3 | | Describe the rationale for the review in the context of what is already known. | | Manuscript page 5 |  |
| Objectives | 4 | | Provide an explicit statement of questions being addressed with reference to participants, interventions, comparisons, outcomes, and study design (PICOS). | | Manuscript page 5 |  |
| **METHODS** | | | | |  |  |
| Protocol and registration | 5 | | Indicate if a review protocol exists, if and where it can be accessed (e.g., Web address), and, if available, provide registration information including registration number. | | Manuscript page 6 |  |
| Eligibility criteria | 6 | | Specify study characteristics (e.g., PICOS, length of follow-up) and report characteristics (e.g., years considered, language, publication status) used as criteria for eligibility, giving rationale. | | Manuscript page 6 |  |
| Information sources | 7 | | Describe all information sources (e.g., databases with dates of coverage, contact with study authors to identify additional studies) in the search and date last searched. | | Manuscript page 6 |  |
| Search | 8 | | Present full electronic search strategy for at least one database, including any limits used, such that it could be repeated. | | Supplementary material – search syntax |  |
| Study selection | 9 | | State the process for selecting studies (i.e., screening, eligibility, included in systematic review, and, if applicable, included in the meta-analysis). | | Manuscript page 6 |  |
| Data collection process | 10 | | Describe method of data extraction from reports (e.g., piloted forms, independently, in duplicate) and any processes for obtaining and confirming data from investigators. | | Manuscript page 7 |  |
| Data items | 11 | | List and define all variables for which data were sought (e.g., PICOS, funding sources) and any assumptions and simplifications made. | | Manuscript page 7 |  |
| **Section/topic** | | **#** | | **Checklist item** | **Reported on page #** | |
| Risk of bias in individual studies | 12 | | Describe methods used for assessing risk of bias of individual studies (including specification of whether this was done at the study or outcome level), and how this information is to be used in any data synthesis. | | Not applicable | |
| Summary measures | 13 | | State the principal summary measures (e.g., risk ratio, difference in means). | | Manuscript page 7 | |
| Synthesis of results | 14 | | Describe the methods of handling data and combining results of studies, if done, including measures of consistency (e.g., I^2^) for each meta-analysis. | | Manuscript page 7 | |

| Risk of bias across studies | 15 | Specify any assessment of risk of bias that may affect the cumulative evidence (e.g., publication bias, selective reporting within studies). | Not applicable |
| --- | --- | --- | --- |
| Additional analyses | 16 | Describe methods of additional analyses (e.g., sensitivity or subgroup analyses, meta-regression), if done, indicating which were pre-specified. | Not applicable |
| **RESULTS** | | |  |
| Study selection | 17 | Give numbers of studies screened, assessed for eligibility, and included in the review, with reasons for exclusions at each stage, ideally with a flow diagram. | Manuscript page 7-8 |
| Study characteristics | 18 | For each study, present characteristics for which data were extracted (e.g., study size, PICOS, follow-up period) and provide the citations. | Table 1 |
| Risk of bias within studies | 19 | Present data on risk of bias of each study and, if available, any outcome level assessment (see item 12). | Not applicable |
| Results of individual studies | 20 | For all outcomes considered (benefits or harms), present, for each study: (a) simple summary data for each intervention group (b) effect estimates and confidence intervals, ideally with a forest plot. | Not applicable |
| Synthesis of results | 21 | Present results of each meta-analysis done, including confidence intervals and measures of consistency. | Manuscript page 8-15 |
| Risk of bias across studies | 22 | Present results of any assessment of risk of bias across studies (see Item 15). | Not applicable |
| Additional analysis | 23 | Give results of additional analyses, if done (e.g., sensitivity or subgroup analyses, meta-regression [see Item 16]). | Not applicable |
| **DISCUSSION** | | |  |
| Summary of evidence | 24 | Summarize the main findings including the strength of evidence for each main outcome; consider their relevance to key groups (e.g., healthcare providers, users, and policy makers). | Manuscript page 15-16 |
| Limitations | 25 | Discuss limitations at study and outcome level (e.g., risk of bias), and at review-level (e.g., incomplete retrieval of identified research, reporting bias). | Manuscript page 22-23 |
| Conclusions | 26 | Provide a general interpretation of the results in the context of other evidence, and implications for future research. | Manuscript page 23 |
| **FUNDING** | | |  |
| Funding | 27 | Describe sources of funding for the systematic review and other support (e.g., supply of data); role of funders for the systematic review. | Manuscript page 24 |
